# How much nicotine kills a human? Tracing back the generally accepted lethal dose to dubious self-experiments in the nineteenth century

**DOI:** 10.1007/s00204-013-1127-0

**Published:** 2013-10-04

**Authors:** Bernd Mayer

**Affiliations:** Department of Pharmacology and Toxicology, Karl-Franzens University Graz, Univ.-Platz 2, 8010 Graz, Austria

The human toxicity of nicotine has become increasingly relevant in the past couple of years through marketing of new nicotine-containing products, such as smokeless tobacco and liquids for electronic nicotine delivery systems (electronic cigarettes) that are freely available in most countries. Standard textbooks, databases, and safety sheets consistently state that the lethal dose for adults is 60 mg or less (30–60 mg), leading to safety warnings that ingestion of five cigarettes or 10 ml of a dilute nicotine-containing solution could kill an adult. The 60-mg dose would correspond to an oral LD_50_ of around 0.8 mg/kg, a dose that is considerably smaller than the values determined for laboratory animals, which are ranging from 3.3 (mice) to more than 50 mg/kg (rats) (Hayes 1982).

Although an LD_50_ of 0.8 mg/kg would implicate that the toxicity of nicotine is similar to or even higher than that of cyanide, fatal nicotine intoxications are relatively rare, and there are countless records of subjects who survived consumption of nicotine in amounts far higher than 60 mg (Larson et al. 1961). The most drastic example is probably survival of a suicide attempt with 4 g of pure nicotine (Schmidt 1931). While this is certainly an exceptional case, in which the amount of bioavailable nicotine was markedly reduced by vomiting, ingestion of tobacco or nicotine gums at doses up to 6 mg/kg nicotine was reported to evoke symptoms of intoxication without causing death (Malizia et al. 1983; Smolinske et al. 1988). These and many other literature reports on nonfatal nicotine intoxications are hardly compatible with a lethal dose of 60 mg or less.

Several detailed reviews are available on fatal nicotine intoxications caused by either suicidal intent or accidents, latter mainly resulting from misusage of nicotine-containing solutions marketed as pesticides (Esser and Kühn 1933; Larson et al. 1961; Maehly and Bonnichsen 1963; Tiess and Nagel 1966; Hayes 1982; Corkery et al. 2010; Solarino et al. 2010). The postmortem data reviewed by Maehly and Bonnichsen (1963) and more recently by Corkery et al. (2010) and Solarino et al. (2010) revealed minimal nicotine blood levels of 2 mg/L, but rapid decline of blood nicotine after death (Sanchez et al. 1996) may have led to underestimation of the actual lethal concentration in delayed autopsies.

Despite these uncertainties and the complex pharmacokinetics of nicotine (Hukkanen et al. 2005), a rough estimate of the amount of ingested nicotine from postmortem analyses of blood levels appears feasible. Smoking a cigarette results in uptake of approximately 2 mg of nicotine and gives rise to mean arterial plasma concentrations of about 0.03 mg/L (30 ng/ml) (Gourlay and Benowitz 1997). Based on 20 % oral bioavailability of nicotine (Hukkanen et al. 2005) and assuming linear kinetics, an oral dose of 60 mg would give rise to a plasma concentration of about 0.18 mg/L. The literature reports on fatal nicotine intoxications suggest that the lower limit of lethal nicotine blood concentrations is about 2 mg/L, corresponding to 4 mg/L plasma, a concentration that is around 20-fold higher than that caused by intake of 60 mg nicotine. Thus, a careful estimate suggests that the lower limit causing fatal outcomes is 0.5–1 g of ingested nicotine, corresponding to an oral LD_50_ of 6.5–13 mg/kg. This dose agrees well with nicotine toxicity in dogs, which exhibit responses to nicotine similar to humans (Matsushima et al. 1995).

The mismatch between the generally accepted lethal dose and documented cases of nicotine intoxication raises the question for the genuine source of the 60-mg dose. Literature and Internet searches provided circular and often misleading references to databases or textbooks, which either simply state the dose without reference or refer to another textbook and so on. To give an example, the following statement is found on the webpage of the Centers for Disease Control and Prevention (http://www.cdc.gov/niosh/idlh/54115.html): *“The fatal human dose has been estimated to be about 50–60* *mg* (*Lazutka* et al. 1969).*”* However, Lazutka et al. describe the determination of LD_50_ values for mice and rats and do not even mention human toxicity (Lazutka et al. 1969). The second paper cited on this Web site (Lehman 1949) was actually published in 1949, and neither provides any supporting data (Lehmann 1949). Screening the German literature published before World War II eventually revealed references to a textbook published in 1906 by Rudolf Kobert (1854–1918), a renowned pharmacologist and pioneer of toxicology in Germany. In the chapter on nicotine of his *Lehrbuch der Intoxikationen*, Kobert makes the following statement (Kobert 1906):

*Vom reinen Nik. ist die lethale Dosis ebenfalls schwer zu bestimmen, da es sich an der Luft leicht etwas zersetzt und andererseits meistens mehr oder weniger wasserhaltig ist; doch nach den üblen Zufällen [sic!], die bei mehreren Experimentatoren schon 0.002–0.004 g hervorbrachten, ist sie wohl sicher nicht höher als 0.06 g*.

English translation (by B.M.)

*The lethal dose of pure nicotine is also difficult to determine, because it easily decomposes a bit and, on the other hand, mostly contains more or less water; however, in accordance with the severe symptoms evoked in several experimenters by 0.002–0.004 g it is certainly not going to be higher than 0.06 g.*

It is beyond any doubt that this short, not particularly convincing paragraph represents the genuine origin of the lethal nicotine dose we still refer to more than 100 years later. What kind of severe symptoms did the experimenters develop that led Kobert to his momentous conclusion? In his book he refers the reader to self-experiments performed by Dworzack and Heinrich, which the Austrian pharmacologist Carl Damian von Schroff (1802–1887) comprehensively described in a Pharmacology textbook (Schroff 1856). Kobert presents a copy of Schroff’s report in condensed and modernized style (Kobert 1906):

*Die Sympt. sind durch Selbstversuche von Reil und später von Dworzack und Heinrich (unter D. Schroff) genau festgestellt worden. Die letztgenannten Autoren empfanden nach 1–4 mg Nik. Brennen im Munde, Kratzen im Rachen, vermehrte Speichelabsonderung, dann vom Magen ausgehend ein Gefühl von Wärme, die sich über die Brust und den Kopf bis in die Zehen- und Fingerspitzen verbreitete. Nachher wurden die Genannten aufgeregt, litten an Kopfschmerz, Schwindel, Betäubung, undeutlichem Sehen und Hören, an Lichtscheu, Beklommenheit, Trockenheit im Schlunde, Kälte in den Extremitäten, Ructus, Flatulenz, Nausea, Erbrechen und Stuhldrang. Die Atmung wurde beschleunigt und angestrengt; die Pulsfrequenz nahm anfänglich zu und zwar umso mehr, je größer die Dosis war; später aber wechselte reglos Zunahme und Abnahme derselben. Nach Verlauf von 45 Minuten wurden die Experimentatoren ohnmächtig. Bei dem einen kam es zu 2 Stunden anhaltenden klonischen Krämpfen, besonders der Atemmuskeln, zu Zittern der Extremitäten und Schütteln des ganzen Körpers. Nach eingetretener Besserung blieb doch Abgeschlagenheit, Schläfrigkeit und trostlose Stimmung noch 3 Tage lang zurück.*

English translation (by B.M.)

*The symptoms were determined exactly in self-experiments by Reil and later by Dworzack and Heinrich (under D. Schroff). After 1–4 mg of nicotine, these authors felt a burning sensation in the mouth, scratchy throat, increased saliva excretion, followed by a feeling of warmness emanating from the stomach, which spread over the chest and from the head to the toes and fingertips. Afterwards the subjects became agitated, suffered from headache, dizziness, numbness, cloudy vision and hearing, light sensitivity, anxiety, dryness of the throat, coldness of the limbs, ructus [belch], flatulence, nausea, vomiting and rectal tenesmus. Respiration was accelerated and labored, pulse rate increased initially, and rose directly with the increasing dose; but later rose and fell erratically. After 45 min the experimenters lost consciousness. One of them suffered clonic seizures for 2 h, particularly of the respiratory muscles, also tremors of the limbs and shivering over the whole body. After the initial recovery, feelings of exhaustion, drowsiness and bleakness remained for 3 days.*

Some of these effects resemble typical symptoms of nicotine overdosing, but 1–4 mg of oral nicotine will certainly not evoke the severe adverse effects described, such as clonic seizures and loss of consciousness. Curiously, Kobert mentioned the Pharmacologist Wilhelm Reil but ignored Reil’s account on very mild symptoms caused by self-ingestion of up to around 7.5 mg of nicotine (15 drops of a solution of 1 drop of nicotine in 100 drops of alcohol) (Reil 1857). Indeed, more recent studies have shown that intravenous administration of up to 5 mg of nicotine, corresponding to 25 mg oral, i.e., 50 % of the allegedly lethal dose, led to only minor adverse effects, such as coughing and nausea (Henningfield et al. 1983; Gourlay and Benowitz 1997). Thus, Kobert estimated the lethal dose of nicotine on the basis of highly dubious self-experiments performed in the mid of the nineteenth century while ignoring conflicting data. His excellent reputation as a leading scholar in toxicology has apparently led to uncritical acceptance and citation of the 60-mg dose by contemporary fellows and successive researchers.

The discrepancy between the 60-mg dose and published cases of nicotine intoxication has been noted previously (Matsushima et al. 1995; Metzler et al. 2005), but nonetheless, this value is still accepted without scrutiny and taken as the basis for worldwide safety regulations of tobacco and other nicotine-containing products. Nicotine is a toxic compound that should be handled with care, but the frequent warnings of potential fatalities caused by ingestion of small amounts of tobacco products or diluted nicotine-containing solutions are unjustified and need to be revised in light of overwhelming data indicating that more than 0.5 g of oral nicotine is required to kill an adult.
